# Neck pain and distance learning: A pain in the neck for university students during COVID-19

**DOI:** 10.12688/f1000research.145874.1

**Published:** 2024-04-22

**Authors:** Awab Musaad Mohamed, Mohamad Abdulkafi Abbara, Sara Alaaeldin Bashier, Danya Aasim Elkhidir, Amal Hussein, Anu Vinod Ranade

**Affiliations:** 1 College of Medicine, University of Sharjah, Sharjah, Sharjah, 27272, United Arab Emirates; 2 Department of Basic Medical Sciences, College of Medicine, University of Sharjah, Sharjah, Sharjah, 27272, United Arab Emirates; 3 Cardiovascular Research Group, Sharjah Institute for Medical Research,, University of Sharjah, Sharjah, Sharjah, 27272, United Arab Emirates

**Keywords:** Neck pain; COVID-19; E- learning; musculoskeletal disorders

## Abstract

**Objective:**

The shift to online learning during COVID-19 led to increased musculoskeletal discomforts and impacted students’ quality of life. Neck pain, once a minor issue, has become more prevalent due to prolonged electronic device use in new learning methods. This study aims to measure the prevalence of neck pain among University of Sharjah (UOS) students during the COVID-19 online learning period and to investigate the factors that provoked it.

**Methods:**

This cross-sectional study used an online survey distributed to UOS students via social media from February 16 to March 12, 2021. Demographic data, Neck Disability Index (NDI) assessments, and pain management information were gathered and analyzed using SPSS 24 through univariate and bivariate methods.

**Results:**

The prevalence of neck pain during COVID-19, among 325 UOS students was found to be 62.7%, 64.41% of which had neck pain at the time of doing the survey. The mean NDI percentage point was 19.19%. The most common location of pain was the back of the neck 38.77%). Participants reported prolonged sitting (81.23%) while leaning forward as the most prevalent cause of their neck pain. Bivariate analysis showed that the most significant factors affecting the NDI were lifting, personal care, headaches, concentration, sleeping, recreational activities, reading, anxiousness, depression, and work. Moreover, subjects who did not seek medical attention treatment had significantly higher NDI scores as compared to those who did (83.89 vs 125.80, p=0.002).

**Conclusion:**

By determining the prevalence of neck pain among UOS students, this research can attract attention to the importance of postural changes and time spent in e-learning on neck pain, and the gaps of previously published articles can be filled. Future studies about neck pain and additional preventative measures should be catalyzed in the UAE.

## Introduction

Neck pain is a common condition that affects a significant proportion of the population. Despite its prevalence, it can have a substantial negative impact people’s lives, including their ability to work, sleep and participate in daily activities. ^1^ The global prevalence of neck pain was 3551.1 per 100 000 people in 2017 with a DALY (disability adjusted life years) prevalence of 352.0 per 100 000 people. ^2^ Chronic neck pain presents a significant burden on societies all over the world. ^3^ Neck pain and other forms of chronic pain in general burdens the workers, increases sickness absence, and decreases productivity. ^4^ Furthermore, it can exacerbate other co-existing conditions such as migraines, ^5^ making it more challenging to manage these conditions effectively. Neck pain may lead to muscle morphology such as hypertrophy or atrophy, alteration in muscle fiber type, and reduced muscle strength. ^6^

In 2018, 440 randomly chosen medical students at Jazan University in Saudi Arabia reported a prevalence of neck pain of 53.5%. ^7^ According to other studies, using laptops and smartphones which many university students depend on all the time, has been identified as a significant risk factor of neck pain. ^8^

Students in the United Arab Emirates (UAE) commonly experience musculoskeletal disorders (MSP) as one of the primary occupational health concerns.

A study conducted in February 2020 among 368 dental students from Ajman University and Ras Al Khaimah College of Dental Sciences reported a prevalence of MSP 48.5% and 68.3% in the past week and year, respectively based on responses to questionnaire. ^9^ The most frequent occupational health hazards faced by newly graduated dentists working in Abu Dhabi, Dubai and Sharjah, as reported in a study conducted on 733 individuals, are musculoskeletal disorders (MSP) at 68% and percutaneous injury at 42%. ^10^

The prevalence of neck pain among 80 radiologists in the United Arab Emirates, comprising of male and female radiologists from nationalities, found a significant rise in the prevalence of neck pain after they started working in the radiology department in 2017. Also, the neck pain surpassed other common work-related symptoms such as back pain, knee pain and vision issues. The researchers speculated that poor body mechanics such as improper posture, repetitive movements and inadequate support may contribute to the development of neck pain. ^11^

The COVID-19 pandemic, which was officially declared on March 11, 2020, has brought about extraordinary challenges in various aspects of life. Outbreaks have led to increased unemployment as well as compromise in education, physical and mental health, resulting in a profound impact on the world. ^12^^,^^13^

Education was one of the fields that has been extremely affected during the COVID-19 pandemic with schools and universities globally have transitioned to distance education. ^14^

Blended and hybrid learning have continued to gain popularity and increase in value beyond the pandemic, mainly due to the necessity for novel and innovative teaching techniques, the potential of cloud-based applications, enhanced collaboration, and creativity. ^15^ Additionally, lack of adherence to good ergonomic practices during online learning at home has led to an increase in reported cases of back pain and fibromyalgia pains. ^16^ Unlike in the classroom, students are not obligated to follow proper ergonomics at home and take online classes while sitting on beds or sofas. These inappropriate prolonged sitting and studying posture have led to the development of musculoskeletal discomfort and complaints including neck pain. ^17^^,^^18^ It is hypothesized that utilizing these devices may cause indirect tension in the neck muscles, eventually leading to neck pain and discomfort. ^6^ According to a Hong Kong research, using electronic devices is associated with musculoskeletal disorders, particularly in the neck and shoulder region. ^11^

Studies have indicated that a lack of knowledge regarding effective strategies for pain management among university students can result in negative consequences for their musculoskeletal pain, leading to a decline in quality of life and potentially significant socioeconomic issues. ^19^ Furthermore, students experiencing musculoskeletal pain, such as low back pain, may have poorer academic performance and higher levels of absenteeism compared to those without pain. ^20^^,^^21^

### Rationale

Although research has investigated the association between neck pain and electronic device use among university students, limited research has focused during the COVID-19 pandemic. University students may be more susceptible to neck pain due to the high demands of their academic work, which often require long periods of concentration in front of electronic devices. Furthermore, it is well-established that poor posture, such as slouching or holding the head in a forward position, can place significant stress on the neck and lead to pain and discomfort. However, despite the high prevalence of neck pain, there is limited research on the factors that may contribute to it, especially among the UAE population, and specifically among university students. Therefore, it is vital to investigate the potential risk factors for neck pain among university students, including the amount of time spent on electronic devices, the type of devices used, and the posture adopted during use.

Such research can provide valuable insights into the prevalence and potential causes of neck pain among this population and help to inform interventions and preventative measures to reduce the incidence of neck pain and improve the overall health and well-being of students.

### Significance

Gaining insight into the factors that increase the likelihood of neck pain in university students can help create interventions such as modifying workstations, increasing breaks, setting reminders to adjust posture, and practicing exercises to improve posture and reduce muscle tension.

### Objectives

• To measure the prevalence of neck pain among UoS students during online learning.
• To identify factors that are associated with the intensity of the neck pain and to evaluate the psychosocial impact related to neck pain (interference of pain with daily activities and obligations, anxiousness, or depression).

## Methods

### Design, population, sampling method and sample size

The study utilized an observational cross-sectional design using STROBE guidelines. The data was collected through questionnaires from a specific target population, which are the UoS students aged between 18-24. The accessible population was limited to students who were available on social media platforms since the questionnaire was posted online. Non-probability volunteer sampling was the chosen sampling method due to the challenges in accessing a vast number of UoS students and young adults during the study period. Moreover, the online questionnaire was disseminated through various social media platforms, and participation was voluntary.

Our sample size (n) was determined to be 400 based on a 50% prevalence (P) and a 5% margin of error (ME), using the formula n=4P(1-P)/SE2.

### Inclusion criteria

All UoS students aged 17-24 studying in the Sharjah campus and able to do the survey.

### Exclusion criteria

Participants who reported neck pain prior to March 7th, 2020, which was determined based on their responses in the questionnaire; those who attributed their neck pain to events unrelated to online learning; individuals with recent trauma or surgery, and those with disabilities.

The expected prevalence of neck pain was set at 50% as no previous studies in the UAE had been found to address the issue. Using 50% as the expected prevalence, the highest possible value, ensured that the minimum required sample size could be achieved. However, the research team ensured that the questionnaire was made available to the largest possible number of individuals to reach the minimum required sample size and to increase the accuracy of the study.

### Data collection

For this study, a self-administered questionnaire was utilized to gather data. It consisted of the Neck Disability Index (NDI) questionnaire, designed to assess the impact of neck pain on daily functioning ^22^^,^^23^ as well as additional questions exploring other factors associated with neck pain. Questionnaire was disseminated across various social media platforms, including WhatsApp, Facebook, Instagram, and others.

Comprising of five sections, the questionnaire consisted of 27 questions covering demographics, pain history, impact on daily activities and mental health, e-learning environment, and management. Most of the questions were close ended, designed to simplify the survey. Nevertheless, three questions were open-ended to provide participants the opportunity to provide comprehensive answers.

Prior to commencing data collection, a pilot study involving 10-15 subjects was carried out in the 2020/2021 academic year. Upon receiving ethical approval, the questionnaire was distributed via social media platforms by each researcher during the spring semester of 2020/2021. Furthermore, the researchers invited their close friends and colleagues to participate, as they were part of the target population.

### Data analysis

SPSS 24 (IBM Corp. Released 2016. IBM SPSS Statistics for Windows, Version 24.0. Armonk, NY: IBM Corp.) was used to analyze the data, employing univariate analysis to generate descriptive statistics, such as frequency and measures of central tendency, as well as bivariate analysis to examine variable relationships. The SPSS license was obtained through the institutional SPSS software license of the University of Sharjah.

Inferential statistical tests, such as Chi-square, t-test, and Pearson correlation, were utilized based on the type of variables, with a significance level set at 5%.

### Ethical consideration

The study obtained ethical clearance from the University of Sharjah research and graduate research ethics committee (REC-21-02-11-02-S) approval date 14.02.2021, with participation being voluntary, anonymous, and risk-free. Data collected were confidential and used exclusively for research purposes.

## Results

Table 1 indicates that a vast majority of the participants, accounting for 98.15%, were University of Sharjah (UoS) students residing in various locations, including different emirates in the UAE and overseas. Of these, 96.31% (313 participants) fell within the age range of the study, which was 18 to 24 years. The prevalence of neck pain among UoS students in general was 62.7%, most participants scored above 60 in the NDI (70.46%) ( Table 2).

**Table 1.** Participants age groups and locations.

Age group	
Less than 18 Years	10
18-24 Years	313
More than 24 Years	2

Location *	
Abudhabi	54
Dubai	55
Sharjah	176
Other Emirates	29
Outside UAE	9

* Location of the student while filling the survey.

**Table 2.** Neck disability index scores.

Score n (%)	
Less than 20	15 (4.61)
21-40	68 (20.92)
41-60	13 (4)
More than 60	229 (70.46)

**Figure 1.**
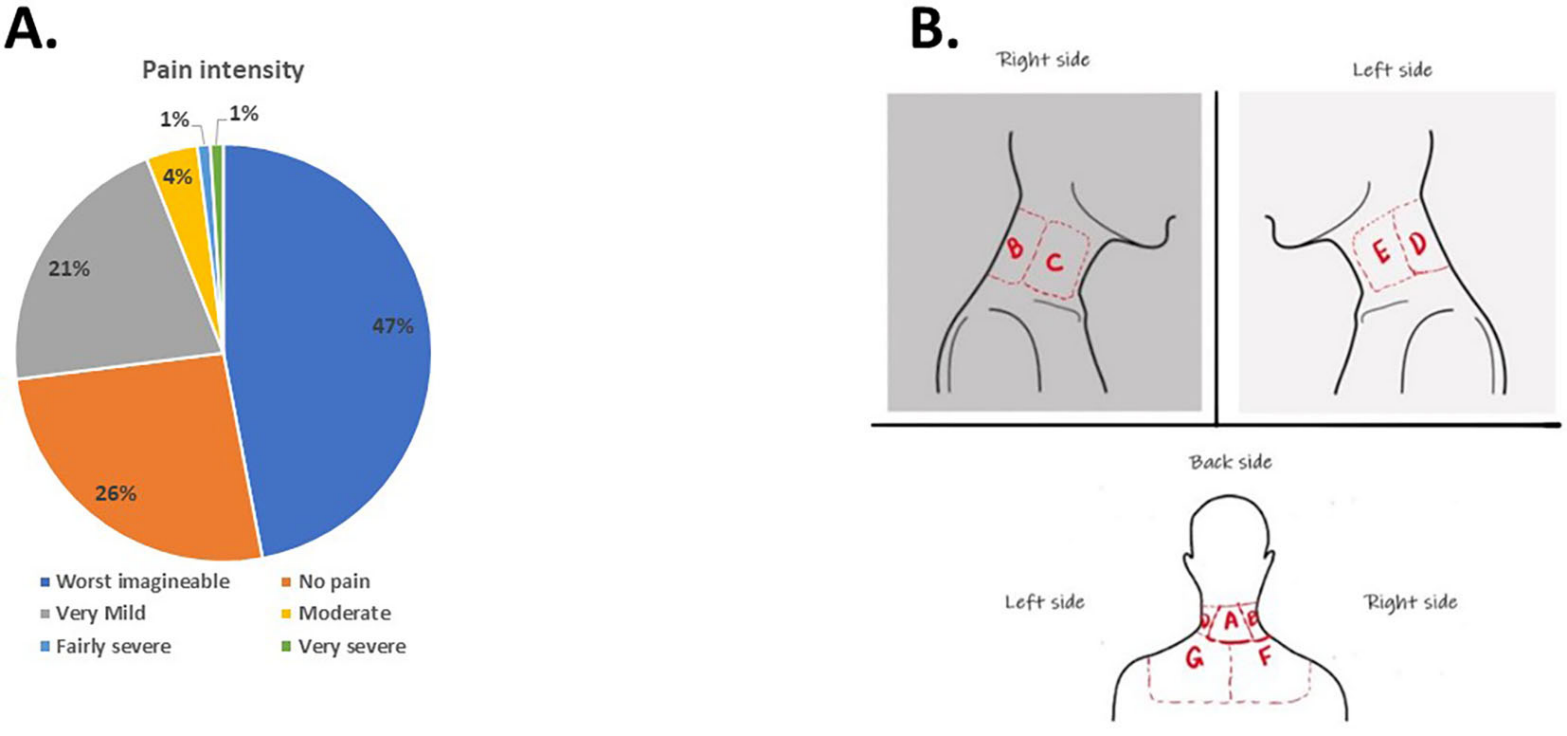
Students’ response to intensity and location of pain.

According to the participants’ responses, the leading causes of neck pain were attributed to incorrect sitting posture and prolonged sitting. Nearly half (47.43%) of the participants experiencing neck pain described it as the worst pain imaginable ( Figure 1A). Regarding the pain location, 38.77% identified location A ( Figure 1B) at the back of the neck as the primary area, while 32.92% chose location F, representing the right lower trapezius, as the source of pain.

**Figure 2.**
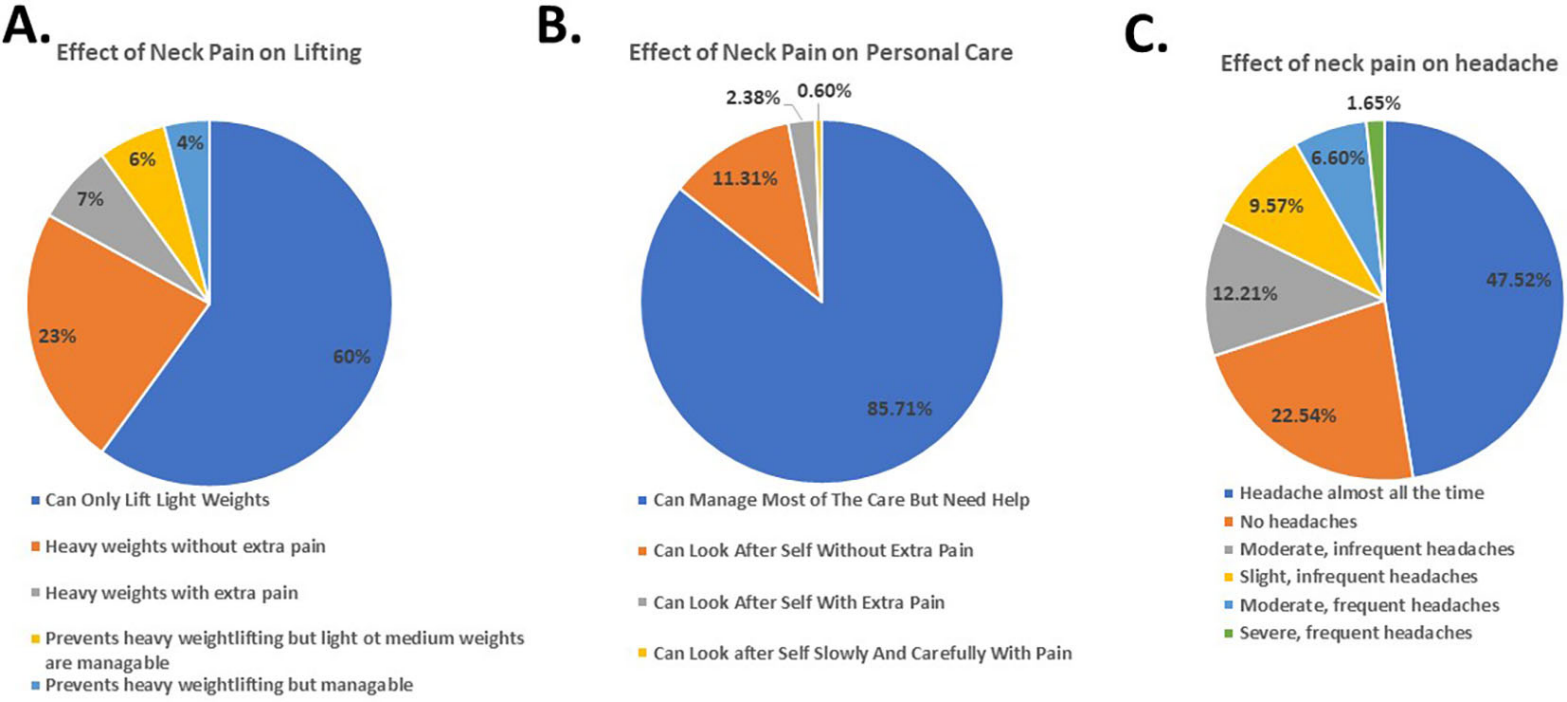
Students’ response to effect of neck pain on lifting weights, personal care and headache.

**Figure 3.**
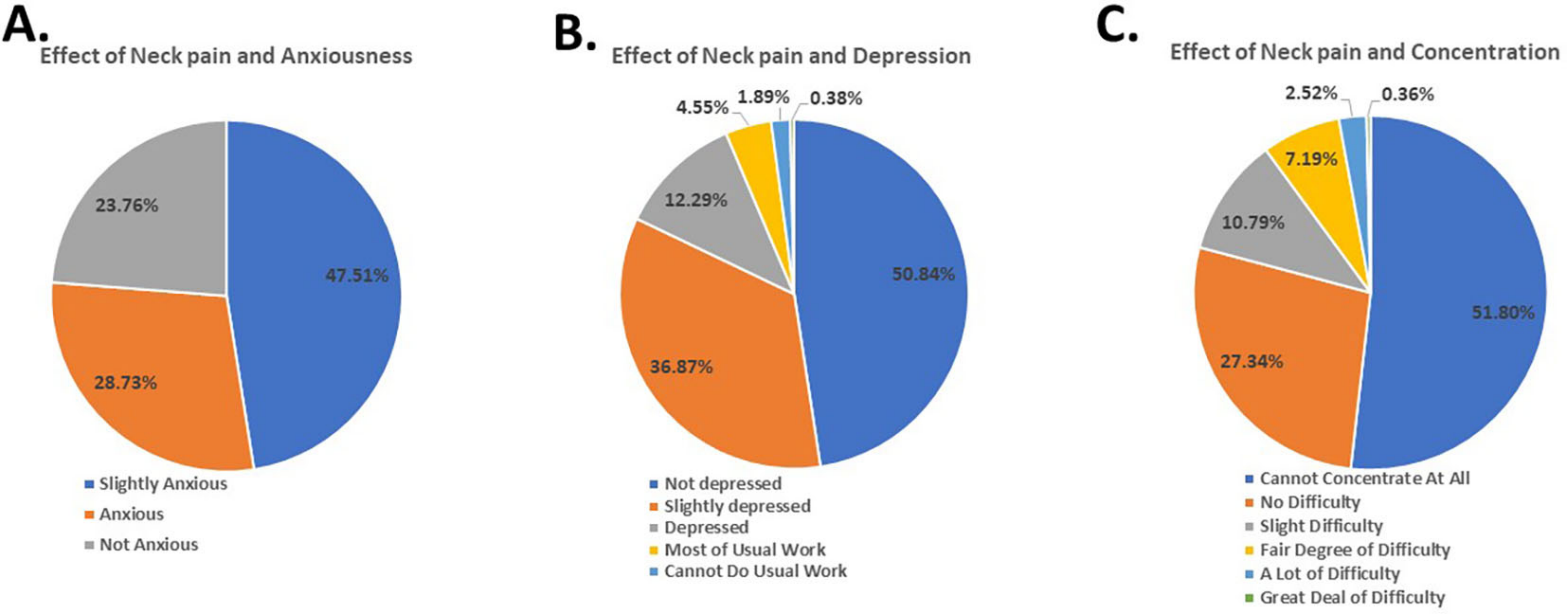
Students’ response to effect of neck pain on anxiousness, depression, and concentration.

According to the Neck Disability Index (NDI) results, a significant majority of participants were able to lift only light weights (60%) ( Figure 2A), needed assistance with personal care (85.71%) ( Figure 2B), and reported having constant headaches (47.52%) ( Figure 2C). Despite feeling slightly anxious due to neck pain (47.51%) ( Figure 3A), the participants did not report experiencing depression because of it (50.84%) ( Figure 3B).

**Figure 4.**
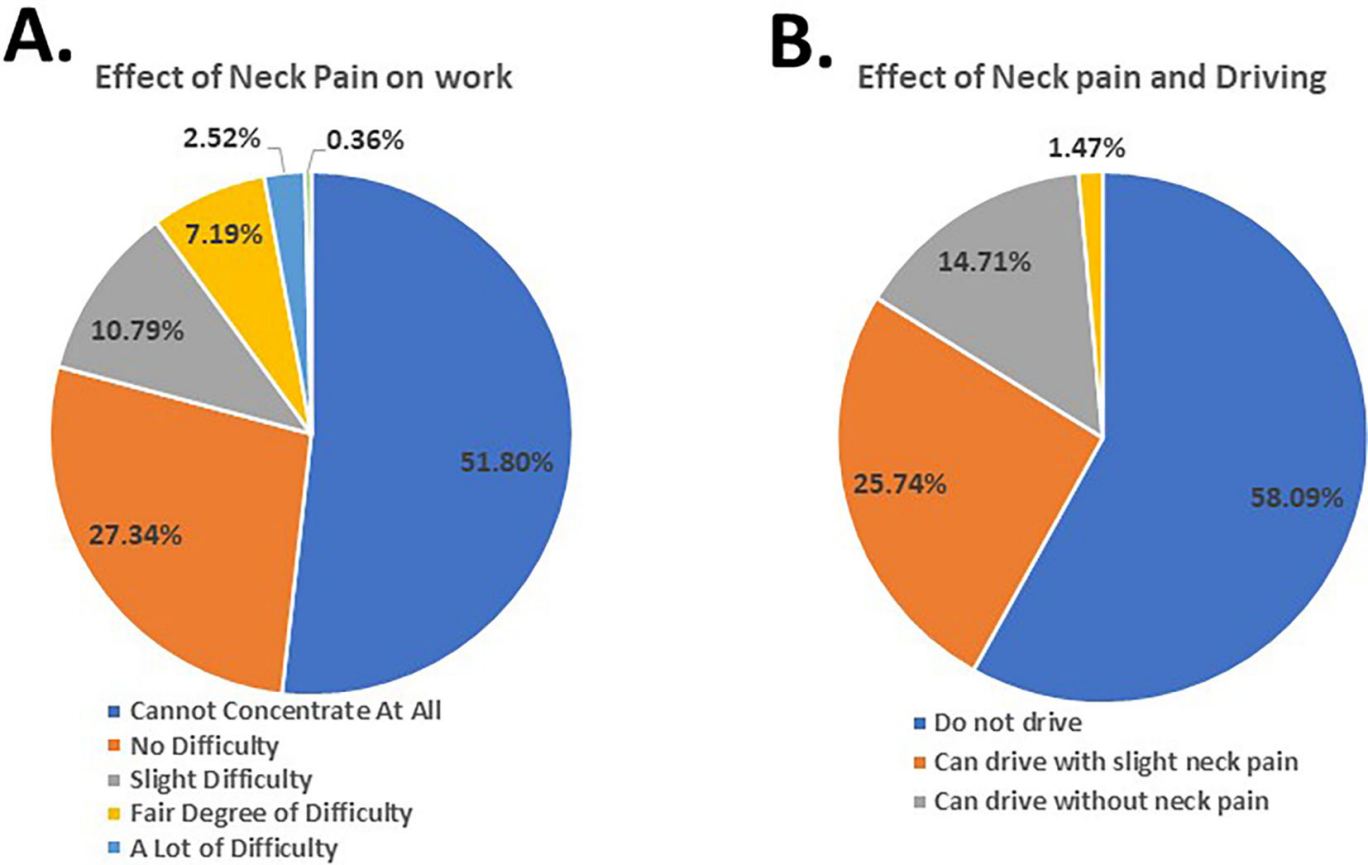
Students’ response to effect of neck pain on work and driving.

**Figure 5.**
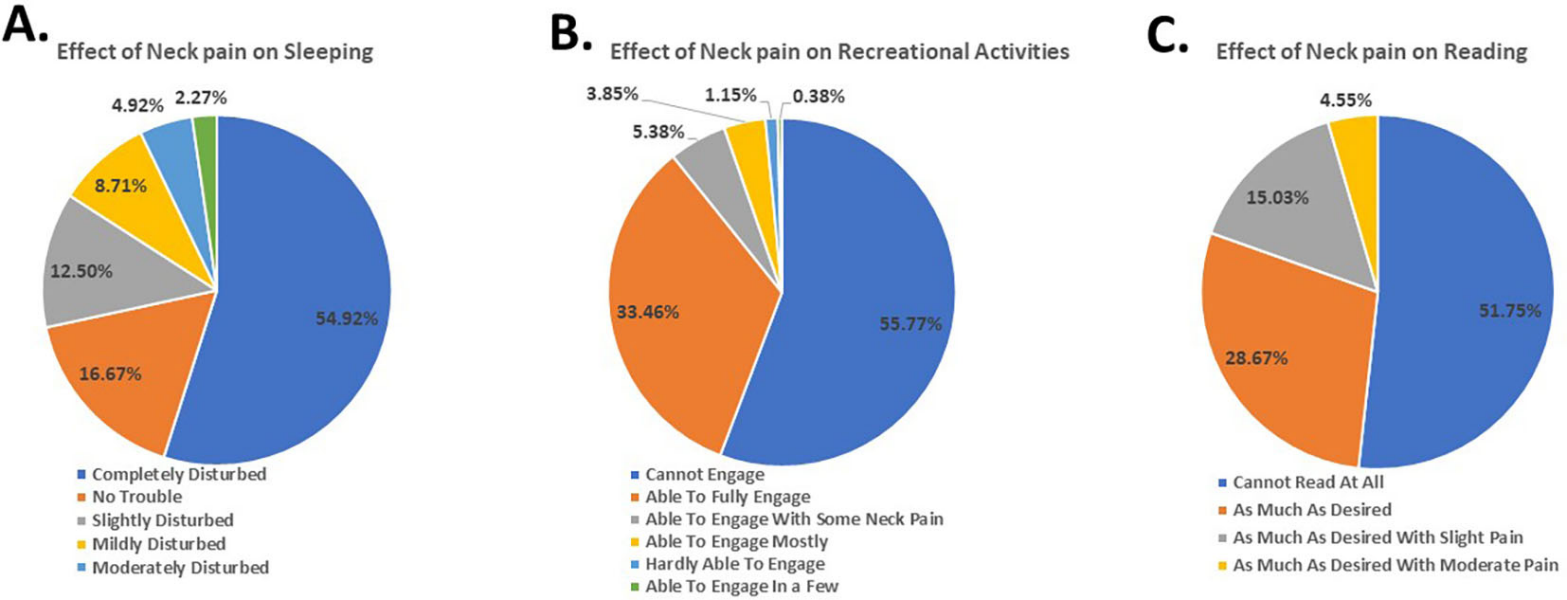
Students’ response to effect of neck pain on sleeping, recreation and reading.

Furthermore, the findings revealed that 51.00% faced difficulty concentrating on their day-to-day activities ( Figure 3C), 54.92% of the participants were unable to work ( Figure 4A), and 25.74% managed to drive their vehicle with slight neck pain ( Figure 4B). Moreover, the participants reported experiencing disturbed sleep (54.92%) ( Figure 5A), difficulty engaging in recreational activities (55.77%) ( Figure 5B), and inability to read (51.00%) ( Figure 5C). These results demonstrate the gravity of the subjects’ neck pain and its impact on their daily routine and tasks.

**Figure 6.**
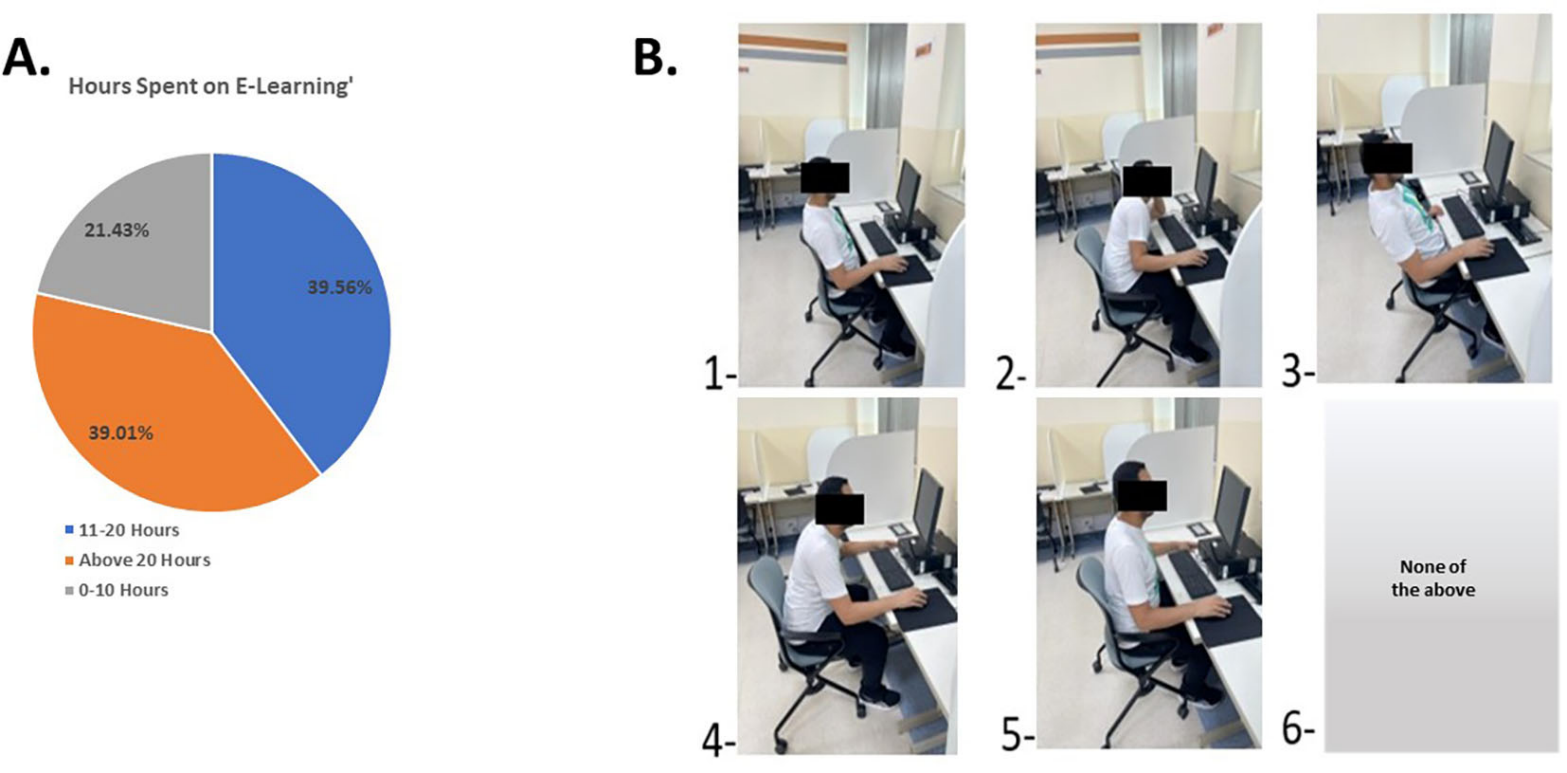
Students’ response to time and the posture used during E-learning.

Regarding the online learning component, 39.56% of participants reported spending 11 to 20 hours per week on online learning ( Figure 6A), with the laptop being the primary device used. The participants’ most common sitting positions ( Figure 6B) were position 2 (32%), where they lean forward onto the device, and position 4 (28%), involving arching the neck and not using the chair to support the back. Positions 1, 3, and 5 were selected by 24%, 23%, and 10% of the participants, respectively.

Merely 32.77% of the participants attempted to mitigate their pain through either medication or exercises (7.38% and 6.77%, respectively). The study revealed that neck pain is often dismissed and considered trivial, as evidenced by 91.48% of the participants not seeking medical assistance to alleviate their pain, and of those who did, only 26.14% were prescribed medication.

The Kruskal-Wallis test results revealed that various lifestyle factors had a substantial impact on the Neck Disability Index (NDI), including lifting, personal care, headaches, anxiousness and depression, work, concentration, sleep, recreational activities, and reading (p>0.005). Notably, driving did not have a significant effect on the NDI score (p=0.186) since most of the participants (58.09%) did not drive. Additionally, participants who did not seek medical attention or receive medical treatment had significant NDI scores (p=0.002).

## Discussion

This study indicates that the prevalence of neck pain among University of Sharjah students during the COVID-19 pandemic-related lockdown period was 62.7%. Of these students, 47.43% experienced neck pain as the worst pain comprehendible. The analysis of the study supports the theory that prolonged sitting for long periods (11-20 hours per week) in front of electronic devices could be a significant cause of neck pain during the unprecedented era of online learning. ^8^^,^^24^^,^^25^ The prevalence of neck pain among undergraduate students in this study is higher than countries, such as Ethiopia (49.2%), ^26^ India (46.9%), ^27^ Singapore (74%) ^28^ and Taiwan (52%) ^29^ and lower than a study conducted in China (72.9%). ^30^ However, the findings is in accord with a study conducted in Brazil (66.7%), ^31^ Pakistan (69%) ^32^ and Malaysia (65.1%). The observed variability in the prevalence rate of NP across studies could be attributed to disparities in multiple factors, including the geographical setting of the study, the size of the sample population, the methodology employed for sampling, and the assessment instruments utilized.

Several studies have established a clear association between the duration of device usage and the occurrence of musculoskeletal pain. ^33^^,^^34^ These findings consistently indicate that as the amount of time spent using devices increases, the risk of developing musculoskeletal injuries also rises. In a study by Berolo et al. (2011) 84% of respondents reported pain in various body regions, with the right hand and thumb being the most frequently affected. ^33^

A study conducted in Australia, among the medical undergraduates, showed neck as the most frequently affected region by musculoskeletal pain. ^35^ Likewise, a study in Thailand reported a significant prevalence of neck pain (46%) among undergraduate students, with various risk factors identified as contributors to persistent neck pain. ^36^ Another study by Alzhrani et al. (2019) 80.1% of participants reported musculoskeletal discomfort, with the most commonly affected areas being the neck (64.7%), back (53.8%), and dominant shoulder (38.8%). ^37^ In similar study by Blair et al. (2015,) 67.9% of participants experienced musculoskeletal symptoms with 70.5% reporting discomfort in the neck (86.4%), lower back (75.9%), and right/left shoulders (76.2%). ^38^ Likewise, Olayinka et al. (2013) found that 75.7% of participants reported shoulder complaints. ^39^ Eugenia et al. (2016) also reported that 49.9% of respondents experienced upper limb musculoskeletal symptoms, particularly in the neck and shoulder regions ^40^ which is comparable to the findings of the present investigation, where 38.77% reported back of the neck and 32.92% right lower trapezius. Extended use of electronic devices can lead to “text neck,” characterized by prolonged neck flexion, which can cause inflammation in neck ligaments, muscles, and nerve irritation. If not addressed, this condition can result in permanent arthritic damage and an increased spinal curvature. ^18^ Our findings add to the growing body of research showing that using electronic devices while studying and adopting bad head position can worsen musculoskeletal pain. ^41^

Also neck pain can significantly disrupt daily activities, potentially hindering individual participation and causing work-related disabilities. Previous reports have conclusively demonstrated the link between neck pain and headaches. ^42^^,^^43^ This study also reveals a simultaneous relationship between activities that induce neck pain, unsteadiness, and headaches among university students. The coexistence of neck pain and headache may stem from the compression of the lesser and greater occipital nerves by posterior cervical muscles, with their fascial attachments at the occipital ridge causing local perineural inflammation. ^44^ Furthermore, this study shows a significant correlation between the neck disability index and various lifestyle factors, including lifting, personal care, recreational activities, in addition to symptoms like anxiety and depression. These factors also impacted the students’ ability to work, perform daily chores, and drive.

Maysoun et al. noted that students using electronic devices in Jordan exhibited severe to extremely severe symptoms of stress, anxiety, and depression, along with moderate to severe insomnia. ^45^ A systematic review and meta-analysis comparing the central processing of pain between individuals with non-traumatic neck pain and healthy subjects revealed that symptoms of depression, which are processed at the spinal, cortical, and brainstem levels, manifest as increased sensitivity to pain in the peripheral regions. ^46^

Additionally, studies from China have indicated that mood disorders are more commonly coexistent with neck pain compared to other mental disorders. Specifically, major depressive disorder exhibited a notably higher association with neck pain than other mood-related conditions. ^47^ This suggests the possibility that the neck pain reported by participants in this study might be linked to mental disorders that emerged during the COVID-19 period.

The rising prevalence of musculoskeletal pain, particularly neck pain among students, calls for urgent preventive measures and proper guidance on posture.

Improper postures like sitting with crossed legs, hunching while walking, and forward neck leaning with rounded shoulders not only lead to neck and shoulder discomfort but can also cause imbalanced spinal pressure, possibly resulting in persistent lower back pain. ^48^^,^^49^

In the present study, most participants leaned forward onto the device or were arching their neck and not using the chair to support the back while sitting.

Effective strategies like exercise can reduce pain intensity, and educational health programs should inform students about the risks of prolonged computer use, though more research is needed to understand pain development and its contributing factors among undergraduates.

This study also provides new insight into the importance of implementing more breaks for neck exercises in between class times, scheduling automatic reminders to adjust postures on all online learning platforms, and apply other standard practices done to manage neck pain like proper positioning of the computers and laptops, as it has been reported to be effective in improving neck function and quality of life. ^50^

Additionally, this study fills in the gaps left by earlier studies that concentrated on either back pain or musculoskeletal pain in general and suggests that future studies concentrate on ergonomic factors like posture and study time, as they believed to be the causes of neck pain according to the students.

The reliability of this data is limited by recall bias. By determining the prevalence of neck pain among UOS students, this research can attract attention to the importance of postural changes and time spent studying on the severity of neck pain, and the gaps of previously published articles can be filled. To further reduce the morbidity of such avoidable pain, additional preventative interventions and future neck pain research should be conducted with relation to the UAE.

## Ethics approval and consent to participate

The study protocol was approved by the University of Sharjah research and graduate research ethics committee Reference number: REC-21-02-11-02-S approval date 14.02.2021), in agreement with accepted international standards.

## Consent for publication

Written consent was taken for publishing the image of the participant.

## Authors’ contribution

**Conceptualization:** Amal Hussein & Anu V Ranade.

**Data curation:** Awab Musaad, Mohamed, Sara Alaaeldin Bashier, Danya Aasim Elkhidir, Mohamad Abdulkafi

**Formal analysis:** Awab Musaad, Mohamed, Sara Alaaeldin Bashier, Danya Aasim Elkhidir, Mohamad Abdulkafi

**Investigation:** Awab Musaad, Mohamed, Sara Alaaeldin Bashier, Danya Aasim Elkhidir, Mohamad Abdulkafi

**Methodology:** Awab Musaad, Mohamed, Sara Alaaeldin Bashier, Danya Aasim Elkhidir, Mohamad Abdulkafi

**Project administration:** Awab Musaad, Mohamed, Sara Alaaeldin Bashier, Danya Aasim Elkhidir, Mohamad Abdulkafi

**Resources:** Awab Musaad Mohamed, Sara Alaaeldin Bashier, Anu V Ranade

**Supervision:** Anu V Ranade & Amal Hussein

**Software:** Awab Musaad Mohamed & Amal Hussein

**Validation:** Amal Hussein & Anu V Ranade

**Visualization:** Amal Hussein & Anu V Ranade

**Writing – original draft:** Awab Musaad, Mohamed, Sara Alaaeldin Bashier, Danya Aasim Elkhidir, Anu V Ranade

**Writing – review & editing:** Anu V Ranade, Awab Musaad Mohamed, Mohamad Abdulkafi

## Data Availability

All data generated and analyzed during this study have been deposited in the Zenodo database. Zenodo: Questionnaire and Data of Neck Pain and Distance learning among university Students During COVID-19, DOI: https://doi.org/10.5281/zenodo.10278960. ^51^ This project contains the following underlying data:
1. Excel for charts.xlsx 2. Questionaire.pdf Creative Commons Attribution 4.0 International
