# Supplementary material for: Neck pain and distance learning: A pain in the neck for university students during COVID-19
Source: F1000Res. 2024 Nov 8;13:307. Originally published 2024 Apr 22. [Version 2] doi: 10.12688/f1000research.145874.2 (PMC11809631; doi:10.12688/f1000research.145874.2)
Supplement: Peer review history

# Reviewer report: Reviewer response for version 2

**DOI:** 10.5256/f1000research.173910.r338881

**Published:** 2024-12-12

**Authors:** Fairus Fariza Zainudin

**Affiliations:** 1 Universiti Pendidikan Sultan Idris, Tanjong Malim, Perak, Malaysia

Accepted the feedback from authors.

Is the work clearly and accurately presented and does it cite the current literature?

Yes

If applicable, is the statistical analysis and its interpretation appropriate?

Partly

Are all the source data underlying the results available to ensure full reproducibility?

Partly

Is the study design appropriate and is the work technically sound?

Partly

Are the conclusions drawn adequately supported by the results?

Partly

Are sufficient details of methods and analysis provided to allow replication by others?

Partly

Reviewer Expertise:

injury pattern and profiling, sports rehabilitation, musculoskeletal pain, sports concussion, Cardiopulmonary resuscitation education.

I confirm that I have read this submission and believe that I have an appropriate level of expertise to confirm that it is of an acceptable scientific standard.

# Reviewer report: Reviewer response for version 2

**DOI:** 10.5256/f1000research.173910.r338883

**Published:** 2024-11-12

**Authors:** B.V. Murlimanju

**Affiliations:** 1 Department of Anatomy, Kasturba Medical College , Manipal Academy of Higher Education, Manipal, Karnataka, India

Dear Editor,

 The authors have revised the manuscript as per the comments. There are few comments which  were not addressed, however they are listed now as the limitations of this study. The changes performed and the scientific contents of this article merit indexing of this article.

Is the work clearly and accurately presented and does it cite the current literature?

Yes

If applicable, is the statistical analysis and its interpretation appropriate?

Yes

Are all the source data underlying the results available to ensure full reproducibility?

Yes

Is the study design appropriate and is the work technically sound?

Yes

Are the conclusions drawn adequately supported by the results?

No

Are sufficient details of methods and analysis provided to allow replication by others?

Yes

Reviewer Expertise:

Morphology, Clinical Anatomy, Neurosurgery

I confirm that I have read this submission and believe that I have an appropriate level of expertise to confirm that it is of an acceptable scientific standard.

# Reviewer report: Reviewer response for version 1

**DOI:** 10.5256/f1000research.159890.r290872

**Published:** 2024-07-22

**Authors:** Fairus Fariza Zainudin

**Affiliations:** 1 Universiti Pendidikan Sultan Idris, Tanjong Malim, Perak, Malaysia

Title:

 It concisely captures the main focus of the study, using appropriate scientific terminology.

 Abstract:

 "Include ethical approval details, participant demographics (age, gender distribution, and faculty representation), and specify whether participants were athletes or non-athletes. Report the mean and standard deviation of NDI scores, both overall and stratified by age groups. Provide information on neck pain severity using validated scales (e.g., VAS or NRS). Briefly describe the methodology for assessing factors affecting neck pain, including questionnaire design. Clarify the NDI scoring system, as the current abstract reports values that exceed 100% (83.89 vs 125.80, p=0.002). In the conclusion, justify the focus on the UAE and articulate the practical implications of this research."

 Results:

 "Address the inclusion of participants under 18 (n=10) and over 24 (n=2) years of age, as these fall outside the stated inclusion criteria. Consider removing these data points and reassessing the findings. Given the established pattern of increasing neck pain prevalence, consider adopting a more stringent significance level (p < 0.025). Provide a detailed explanation of the NDI scoring system and severity classification, citing relevant normative data. Elucidate the process for selecting neck pain-related factors during the pandemic. Report the results of pilot testing and tester reliability. In Table 2, include mean and standard deviation for NDI scores."

 Discussion:

 "Revise the statement on 'rising prevalence' to reflect the cross-sectional nature of this study, acknowledging that trend analysis would require longitudinal data from multiple institutions. Elaborate on the biomechanical and ergonomic factors contributing to prolonged poor posture among students. Reconsider the phrase 'new insight' regarding the importance of breaks for neck exercises, as this is a well-established principle in ergonomics and occupational health. Deepen the discussion by incorporating physiological, behavioral, biomechanical, and psychological perspectives when comparing results to existing literature. Enhance the practical implications section to provide clear, actionable recommendations based on the study findings."

Is the work clearly and accurately presented and does it cite the current literature?

Yes

If applicable, is the statistical analysis and its interpretation appropriate?

Partly

Are all the source data underlying the results available to ensure full reproducibility?

Partly

Is the study design appropriate and is the work technically sound?

Partly

Are the conclusions drawn adequately supported by the results?

Partly

Are sufficient details of methods and analysis provided to allow replication by others?

Partly

Reviewer Expertise:

injury pattern and profiling, sports rehabilitation, musculoskeletal pain, sports concussion, Cardiopulmonary resuscitation education.

I confirm that I have read this submission and believe that I have an appropriate level of expertise to confirm that it is of an acceptable scientific standard, however I have significant reservations, as outlined above.

# Response

**Published:** 2024-10-23

**Authors:** Anu V

**Affiliations:** Basic Sciences, University of Sharjah, Sharjah, Sharjah, United Arab Emirates

Abstract:

1. Reviewers comment: "Include ethical approval details, participant demographics (age, gender distribution, and faculty representation), and specify whether participants were athletes or non-athletes.
  Response: Included in the manuscript as suggested. (This cross-sectional study used an online survey distributed to University of Sharjah students via social media from February 16 to March 12, 2021, following ethical approval. The study participants were young adults aged 18 to 26. Due to the predominantly female demographic of the university student population and pandemic-related restrictions, gender and athletic status were not considered significant variables in this research.)
2. Reviewers comment:  Report the mean and standard deviation of NDI scores, both overall and stratified by age groups.
  Response: Included in the manuscript as suggested ("Participants who did not seek medical attention had significantly higher levels of neck pain disability, as measured by the NDI. The median NDI score for those who did not seek treatment was 20, with an interquartile range of 16.89. In contrast, the median NDI score for those who did seek medical attention was 34.00, with an interquartile range of 22.")
3. Reviewers comment: Provide information on neck pain severity using validated scales (e.g., VAS or NRS). Briefly describe the methodology for assessing factors affecting neck pain, including questionnaire design.
  Response: Since our objective was to identify factors that are associated with the intensity of the neck pain rather than the intensity itself. The question in the questionnaire was added to get a subjective view of how participants express their neck pain intensity.
4. Reviewers comment: Clarify the NDI scoring system, as the current abstract reports values that exceed 100% (83.89 vs 125.80, p=0.002). In conclusion, justify the focus on the UAE and articulate the practical implications of this research. 
  Response: Thank you for bringing this to our notice. The mean rank was reported by mistake. Since the data is skewed in this question, the median and interquartile range were added. Justifications were added to the conclusion as requested.

Results:

1. Reviewers comment: "Address the inclusion of participants under 18 (n=10) and over 24 (n=2) years of age, as these fall outside the stated inclusion criteria. Consider removing these data points and reassessing the findings. Given the established pattern of increasing neck pain prevalence, consider adopting a more stringent significance level (p < 0.025). Provide a detailed explanation of the NDI scoring system and severity classification, citing relevant normative data. Elucidate the process for selecting neck pain-related factors during the pandemic. Report the results of pilot testing and tester reliability. 
  Response: Included as suggested. (NDI scores were found to be skewed. The median is 16% which indicates mild neck pain. The interquartile range is 19.11. Participants who did not seek medical attention had significantly higher levels of neck pain disability, as measured by the NDI. The median NDI score for those who did not seek treatment was 20, with an interquartile range of 16.89. In contrast, the median NDI score for those who did seek medical attention was 34.00, with an interquartile range of 22).
2. Reviewers comment:  In Table 2, include mean and standard deviation for NDI scores.
  Response: As stated earlier, the NDI scores were found to be skewed. The median and interquartile range were added to table 2. 

Discussion:

1. Reviewers comment: "Revise the statement on 'rising prevalence' to reflect the cross-sectional nature of this study, acknowledging that trend analysis would require longitudinal data from multiple institutions.
  Our response: Modified as in the manuscript as suggested (The observed prevalence of musculoskeletal pain, particularly neck pain, among students in this cross-sectional study necessitates further investigation. Longitudinal data from multiple institutions are essential to establish definitive trends and inform comprehensive interventions.)
2. Reviewers comment: Elaborate on the biomechanical and ergonomic factors contributing to prolonged poor posture among students. Reconsider the phrase 'new insight' regarding the importance of breaks for neck exercises, as this is a well-established principle in ergonomics and occupational health. 
  Our response: Modified in the manuscript as suggested (The excessive use of electronic devices can lead to "text neck," a condition characterized by chronic neck flexion. This can result in inflammation of the neck's ligaments, muscles, and nerves, potentially leading to long-term consequences such as permanent arthritic damage and an increased spinal curvature. Biomechanical factors, such as repetitive strain on neck muscles from prolonged forward head posture (Goodarzi et al., 2018), and ergonomic factors, such as the use of poorly designed furniture and inadequate lighting (Eldho & Muthukumar, 2022; Meshram et al., 2021; Brewer, 2006), can contribute to poor posture and exacerbate the development of text neck. These combined factors can increase the risk of musculoskeletal pain and discomfort among students. While the efficacy of incorporating neck exercise breaks is well-established in ergonomics and occupational health literature, this study further underscores its significance in mitigating the negative effects of prolonged sedentary behavior on student health. In addition to regular breaks, implementing strategies such as scheduling automatic reminders to adjust posture on online learning platforms and adopting proper computer and laptop positioning can significantly enhance neck function and quality of life. [50]. These interventions, when combined with neck exercises, can effectively alleviate NP and discomfort among students.)
3. Reviewers comment: Deepen the discussion by incorporating physiological, behavioral, biomechanical, and psychological perspectives when comparing results to existing literature. Enhance the practical implications section to provide clear, actionable recommendations based on the study findings." 
  Our response: Incorporated in the manuscript as suggested. (The development of NP is influenced by a multifaceted interplay of factors. Demographic factors, such as age, gender, height, and weight, can predispose individuals to NP. Biomechanical factors, including forceful exertions, repetitive movements, and awkward postures, can exacerbate existing conditions or contribute to the development of new ones. Psychosocial factors, such as job stress, work dissatisfaction, and poor ergonomic practices, and behavioral factors, such as tobacco use, alcohol consumption, physical activity levels, stress management, and sleep hygiene, can all contribute to the risk of NP (McLean SM et al., 2010). Additionally, environmental factors, such as the physical layout of a workplace or the nature of tasks performed, can influence the likelihood of its occurrence (Kanchanomai et al., 2011; Razvi et al., 2018; Kalirathinam et al., 2017; Lee et al., 2018).Biomechanical factors, such as repetitive strain on neck muscles from prolonged forward head posture (Goodarzi et al., 2018), and ergonomic factors, such as the use of poorly designed furniture and inadequate lighting (Eldho & Muthukumar, 2022; Meshram et al., 2021; Brewer, 2006), can contribute to poor posture and exacerbate the development of text neck. These combined factors can increase the risk of musculoskeletal pain and discomfort among students. University students frequently experience significant perceived stress, particularly in the form of academic stressors. These stressors can include academic demands, financial concerns, time pressures, health issues, and self-imposed pressures (Pamela, L. 2009). Studies have linked high levels of mental stress among students to an increased risk of musculoskeletal disorders, such as low-back pain and neck pain (Lundberg U, et al., 2002). Additionally, research has demonstrated that time pressure can increase the activity of various muscles, potentially leading to increased biomechanical load and musculoskeletal disorders. For example, studies have shown that time pressure can increase the activity of the trapezius, infraspinatus, deltoid, and extensor digitorum muscles (Birch et al., 2003), which can contribute to strain and discomfort in the neck, shoulders, and upper back. 
  Conclusion: This study underscores the prevalence of NP among University of Sharjah students during the COVID-19 pandemic. Prolonged electronic device use and poor posture were identified as primary contributors. Given the limited local research in this area, the study focused on the United Arab Emirates. The findings highlight the necessity for implementing preventive measures, raising awareness, and establishing regulations to mitigate the adverse health effects of distance learning. These measures should include promoting proper posture, encouraging regular breaks, and providing educational resources. Further research is imperative to address this emerging health concern.)

# Reviewer report: Reviewer response for version 1

**DOI:** 10.5256/f1000research.159890.r290876

**Published:** 2024-07-04

**Authors:** Tomas Nakazato

**Affiliations:** 1 School of Medicine, Universidad de Piura, Lima Campus, Lima, Peru

The objective of the study is remarkably interesting and worthy, and I appreciate the effort the authors have put into this work; however, I have several observations and found many fundamental flaws.

 Among the several observations and shortcomings, I would like to highlight some of the most noticeable ones for me.

 INTRODUCTION

1. Authors do not use abbreviations as customary. For example, neck pain may be NC, to avoid repetition and to improve clarity and conciseness of the paper.
2. Some paragraphs are very short; they could be merged for better redaction.
3. Many references discuss musculoskeletal pain in general, rather than specifically focusing on neck pain (Ref. 9, 10, 11, 16, 17, 18, 19, 20), and not related to university students or the young population.

METHODS.

1. The study aims to measure the prevalence of neck pain among University of Sharjah (UOS) students during the online learning period for the COVID-19 pandemics and to investigate the factors that caused it. In our opinion, the method to obtain the prevalence was not appropriate, since the authors used a questionnaire posted online on different platforms  (Facebook, WhatsApp, Instagram, and “others”). We do not know if the 'others' social media platforms are reliable to collect information.
2. As participants were volunteers, we consider that people with neck pain were more likely to participate than healthy individuals, increasing the sampling bias of neck pain conditions.
3.  As researchers called their close friends and colleagues to participate, it may increase the response bias of the study.
4. There is no definition of 'neck pain': the main variable. There is a huge difference between acute and chronic neck pain, neck pain that radiates to the upper limb, neck pain that  radiates to the head, etc. Acute neck pain usually resolves spontaneously and does not condition disability in most cases. Radiculopathic neck pain is very different from non-specific neck pain, and so on.
5. The target population was first defined as UoS students aged 18-24. Then, the inclusion criteria comprised all UoS students aged 17-24 studying at the Sharjah campus. Subsequently, in the results authors accounted that 98.15% were UoS students (so 1.85% were not UoS students? And if that was the case, why did the authors include these data?).
6. The authors used a self-administered questionnaire. The problem we see is that it consisted of a "modified" Neck Disability Index (NDI), adding additional questions to explore other factors associated with neck pain. We should be very careful if we modify standardized instruments to collect information. We do not know whether this modified NDI was validated. Furthermore, we do not know if it was administered in English or the official language of the UAE. There is a reference (23) on the validation of the Neck Disability Index in Serbian patients and another on the comparison of the NDI vs. other pain questionnaires (22), both of which do not clarify our doubts. These may result in improper answers from participants.
7. In the questionnaire, the authors try to explore the effects of neck pain on the anxiousness and depression of participants. In our opinion, we do not agree to explore complex conditions such as anxiousness and depression with two simple questions. If self-reported, these conditions should be explored with standardized questionnaires evaluating different aspects to conclude if the participant has some degree of anxiousness, anxiety and/or depression in the survey.
8. We checked the drawing to detect the location of the pain in the original questionnaire, which was different from that presented in the manuscript. In our view, the original drawing was not suitable for the appropriate location of neck pain by the participants.

RESULTS

1. For the reasons mentioned above, we have doubts about many of the results presented in this study. For example, it is very difficult for us to accept that nearly half of the participants experienced neck pain described as the ' *worst* *pain* imaginable' (47.43%), more than half of the participants ´could not concentrate *at* *all*´ (51.80%) or had ' *completely* disturbed sleep' (54.92%). These results mean that half of the students were unable to study or even function at all, which seems unrealistic to us. Furthermore, with that level of pain and disability reported, in our opinion, it is not conceivable that 91.48% of participants did not seek medical assistance (most individuals with that level of compromise should have sought at least an online medical consultation, according to our clinical practice). In addition, using the same logic, it is inconceivable that only 32.77% of cases tried to alleviate their pain.
2. “The Kruskal-Wallis test results revealed that various lifestyle factors had a substantial impact on the Neck Disability Index (NDI), including lifting, personal care, headaches, anxiousness and depression, work, concentration, sleep, recreational activities, and reading”. To our knowledge, NDI does not measure anxiousness or depression. This modification was apparently made by the authors and should have been shown separately.
3. We believe that the figures are not presented properly. For example, we do not understand the criterion why Figures 2, 3, 4, and 5 are grouped separately with pie charts named A, B, and C. If results are part of a standardized questionnaire (NDI Score), graphics from this source of data should be presented as a whole (an individual figure with pie charts).

DISCUSSION

1. Discussion does not highlight the (several) limitations of the study. The only limitation presented was the recall bias.
2. The comparison of the prevalence of neck pain in this study with other countries is inaccurately presented.
3. Same observation as in the Introduction: Some paragraphs are short; they could be merged for better redaction.

CONCLUSION

1. The conclusion does not reflect the results of the study, but rather contains comments or opinions from the authors.

Statistical analysis and its interpretation:

 Based on numerous observations, we deemed a review of statistical analysis and interpretation unnecessary at this time.

Is the work clearly and accurately presented and does it cite the current literature?

Partly

If applicable, is the statistical analysis and its interpretation appropriate?

I cannot comment. A qualified statistician is required.

Are all the source data underlying the results available to ensure full reproducibility?

Yes

Is the study design appropriate and is the work technically sound?

No

Are the conclusions drawn adequately supported by the results?

No

Are sufficient details of methods and analysis provided to allow replication by others?

Partly

Reviewer Expertise:

Physical Medicine and Rehabilitation. Musculoskeletal Pain.

I confirm that I have read this submission and believe that I have an appropriate level of expertise to state that I do not consider it to be of an acceptable scientific standard, for reasons outlined above.

# Reviewer report: Reviewer response for version 1

**DOI:** 10.5256/f1000research.159890.r275293

**Published:** 2024-05-25

**Authors:** B.V. Murlimanju

**Affiliations:** 1 Department of Anatomy, Kasturba Medical College , Manipal Academy of Higher Education, Manipal, Karnataka, India

This manuscript is very interesting and offers a novel information to the scientific community. I could suggest only few changes.

 1. The subheadings in the background/introduction could be removed.

 2. The recent articles about the ergonomics of a university students can be cited.

 3. Any details of the doctors consultation, radiographs, MRI neck taken, usage of cervical collar can be added in the results section.

 4. If not advised/used, this can be mentioned.

 5. The comparison between male and female need to be added.

 6. The conclusion section to be included. This can be a reflection of the objectives.

Is the work clearly and accurately presented and does it cite the current literature?

Yes

If applicable, is the statistical analysis and its interpretation appropriate?

Yes

Are all the source data underlying the results available to ensure full reproducibility?

Yes

Is the study design appropriate and is the work technically sound?

Yes

Are the conclusions drawn adequately supported by the results?

No

Are sufficient details of methods and analysis provided to allow replication by others?

Yes

Reviewer Expertise:

Morphology, Clinical Anatomy, Neurosurgery

I confirm that I have read this submission and believe that I have an appropriate level of expertise to confirm that it is of an acceptable scientific standard, however I have significant reservations, as outlined above.

# Response

**Published:** 2024-10-23

**Authors:** Anu V

**Affiliations:** Basic Sciences, University of Sharjah, Sharjah, Sharjah, United Arab Emirates

1. Reviewers comment: The subheadings in the background/introduction could be removed.
  Our response: Removed as suggested
2. .Reviewers comment: The recent articles about the ergonomics of a university students can be cited. 
  Our response: Articles related to ergonomics of a university students are included in the manuscript as per the reviewer’s suggestion. (Enhancing student awareness of postural changes and risk factors, coupled with the implementation of ergonomic practices, can significantly mitigate musculoskeletal symptoms, including neck pain (Kilroy, et al., 2000; Bansode et al., 2016; Naik Prashant et al., 2017)).
3. Reviewers comment: Any details of the doctor’s consultation, radiographs, MRI neck taken, usage of cervical collar can be added in the results section. If not advised/used, this can be mentioned. 
  Our response: Mentioned in the manuscript as suggested (Since the study did not include information regarding the doctor's consultation, radiographs, MRI neck, or the use of a cervical collar, we have incorporated the same in the results section.
4. Reviewers comment: The comparison between male and female needs to be added. 
  Our response: Thank you for raising this important comment. We agree that a comparison between male and female students would have been necessary to investigate whether sex is a factor associated with the prevalence of neck pain and/or its intensity. Yet, as related to the context of this study, knowing that the majority of the student population at the University of Sharjah, from which the study sample was selected, includes females, thus the variation concerning sex distribution is minimal. Sex was not among the measured variables and therefore, conducting this comparison will not be possible at this point due to data anonymity. In response to the reviewer's comment, we have added a statement under the study limitations to reflect this gap. (Limitations: This study is subject to several limitations. Firstly, self-selection bias may have influenced the results, as individuals with neck pain may be more likely to participate in an online survey than those without symptoms. This could potentially contribute to the observed high prevalence of neck pain in the study population. Secondly, the targeted recruitment of University of Sharjah students, friends, and colleagues may have introduced response bias. Lastly, in this study, comparing the prevalence of neck pain between male and female students was not done due to the lack of sufficient diversity in the study population with respect to sex distribution. Therefore, we recommend future research to investigate this association.)
5. Reviewers comment: The conclusion section to be included. This can be a reflection of the objectives. 
  Our response: Included in the manuscript as suggested (This study highlights the significant prevalence of neck pain among University of Sharjah students during the COVID-19 pandemic. Prolonged electronic device use and poor posture were identified as primary causes. The findings underscore the need for preventive measures, including promoting proper posture, encouraging regular breaks, and providing educational resources. Further research is necessary to address this growing health concern.)
